# Evaluation of team-based learning in a doctor of physical therapy curriculum in the United States

**DOI:** 10.3352/jeehp.2017.14.3

**Published:** 2017-02-28

**Authors:** Donald H. Lein Jr., John D. Lowman, Christopher A. Eidson, Hon K. Yuen

**Affiliations:** 1 Department of Physical Therapy, School of Health Professions, The University of Alabama at Birmingham, Birmingham, Ala., USA; 2 Department of Occupational Therapy, School of Health Professions, The University of Alabama at Birmingham, Birmingham, Ala., USA; Hallym University, Korea

**Keywords:** Health occupations, Physical therapists, Students, Education, Professional, Educational measurement

## Abstract

**Purpose:**

The purpose of this retrospective study was to evaluate students’ academic outcomes after implementation of the team-based learning (TBL) approach in patient/client management courses in an entry-level doctor of physical therapy (DPT) curriculum.

**Methods:**

The research design of this study involved comparing written and practical exam scores from DPT student cohorts taught with the traditional instructional methods (lecture-based) to those of students from subsequent cohorts taught using the TBL approach in two patient/client management courses: basic skills and cardiopulmonary. For this comparison, the exams used, the number of contact hours and labs, and the instructors who taught these courses remained the same during the transition between these two instructional methods (traditional vs. TBL). The average of all individual course exam scores was used for data analysis.

**Results:**

In both courses, there were no meaningful differences in the mean exam scores among students across years of cohorts receiving the same instructional method, which allowed clustering students from different years of cohorts in each course receiving the same instructional method into one group. For both courses, the mean exam score was significantly higher in the TBL group than in the traditional instruction group: basic skills course (P<0.001) and cardiopulmonary course (P<0.001).

**Conclusion:**

Student cohorts taught using the TBL approach academically outperformed those who received the traditional instructional method in both entry–level DPT patient/client management courses.

## Introduction

Team-based learning (TBL) is an active teaching/learning technique that promotes individual and group accountability, collaborative learning, and acquisition of higher-order cognitive skills [1]. All of these skills are essential for health professionals in today’s healthcare environment [2]. Improvements in students’ knowledge scores resulting from the implementation of TBL in health professions education have been well documented [3]. Despite the potential benefits of TBL, the only published literature regarding TBL in doctor of physical therapy (DPT) curricula, to our knowledge, is in a foundational science course, gross anatomy [4-6]. In these studies, investigators compared academic performance after receiving TBL with dissection lab or lecture with dissection lab. The purpose of this study was to evaluate the students’ academic outcomes after implementation of the TBL approach in patient/client management courses in an entry-level DPT curriculum.

## Methods

### Study design

This retrospective study compared the academic outcomes of different entry-level DPT student cohorts before and after implementation of TBL in two patient/client management courses.

### Doctor of physical therapy academic program

The DPT academic program at the University of Alabama at Birmingham (UAB) is a 3-year (9-semester) program. Physical therapy (PT) intervention I (basic skills) is a 3-credit course offered in the 1st semester and focuses on the following intervention skills: gait training with assistive devices, transfers, superficial modalities, mechanical modalities, standard and special precautions, documentation, basic communication, massage, and positioning. PT management of cardiopulmonary dysfunction (cardiopulmonary) is a 3-credit course offered in the 5th semester (i.e., 2nd year in the curriculum) and focuses on examination, evaluation, and interventions for patients/clients with primary or secondary cardiovascular or pulmonary impairments.

### Student cohorts

For the basic skills course, exam scores were available for one student cohort (2011) receiving traditional instructional method (lecture-based with labs) and three cohorts (2012-2014) receiving the TBL approach with labs. Although the TBL approach was still used in the basic skills course, the course content was altered in 2015 and, therefore, exam scores for 2015 and 2016 were not included for analysis. For the cardiopulmonary course, exam scores for four cohorts (2006, 2007, 2009, and 2011) receiving traditional instructional method (lecture-based with labs), and five cohorts (2012-2016) receiving the TBL approach with labs were available. The grading criteria for the cardiopulmonary course were altered in 2008 and 2010 such that numerical scores were not available for analysis. Course content/objectives, instructors, lab/class time, and written/practical exams remained the same within each course across all cohorts, before and after adoption of TBL.

### Participants

The number of students enrolled (i.e., student cohort) in each of these courses varied from year to year, ranging from 31 to 50. The student cohort enrolled in year one for the basic skills course were the same students in the cardiopulmonary course the subsequent year, although there was frequently minimal attrition. Number of students in each year’s cohort for each course is shown in Table 1. Team assignment for students, discussed below, remained the same in courses taught with TBL throughout the curriculum.

### Ethical approval

The UAB institutional review board approved the study protocol (X120206009).

### Team-based learning process

Each of the patient/client management courses consisted of several modules. Through the TBL process, students experienced the same sequence for each module, including (1) advanced preparation (step 1), (2) readiness assurance (steps 2-5), and (3) application of course concepts (step 6) [7].

*Step 1 (individual study)*: Students completed pre-class assigned readings/independent learning activities based on module objectives.

*Step 2 (individual test)*: Students took a multiple-choice format readiness assurance test (RAT) that covered basic concepts of the assigned readings (Step 1). The individual RAT (iRAT) was administrated with one item at a time projected on the screen. Students registered their answer using an audience response system (i>clicker, http://www1.iclicker.com). This system gives the instructor immediate feedback on class performance for each item.

*Step 3 (team test)*: Each team was given a team RAT (tRAT) which was a paper copy with the exact same test questions that were on the iRAT. Intra-team discussion led to a consensus answer for each item and student teams registered their answer via an immediate feedbackassessment technique form (IF-AT, http://epsteineducation.com), a ‘scratch off’ answer sheet that provided the team with confirmation of a correct response (and additional tries if an incorrect response is selected).

*Step 4 (written appeals)*: Since the team knew whether or not they got an item correct (from the IF-AT form), team appeals were promoted by the instructor for any items missed. This open-book process encouraged students to look up items they answered incorrectly and articulate in writing why they selected a different option.

*Step 5 (instructor input)*: The instructor then provided a ‘minilecture’ that covered concepts/materials missed by the majority of the students on the iRAT and new concepts/materials not covered in the assigned readings but deemed important.

*Step 6 (team application activities)*: The application activities consisted of patient case studies with multiple-choice test items based on these cases. These items required higher order thinking skills than those in the RAT. Each team worked on the same case and answered the corresponding question(s); this process further promoted intrateam discussion over clinically relevant issues. Teams then simultaneously reported their answer for each item and any differences between teams were discussed in class, promoting inter-team dialogue. Misconceptions were clarified by other teams and/or the instructor [1,7].

Prior to the first TBL session, the instructors created teams with 7 to 8 members per team. To facilitate balanced and diverse member composition in teams, a variety of baseline, individual student characteristics were considered: gender, race/ethnicity, undergraduate major and college attended, undergraduate grade point average (UGPA), graduate record examination (GRE) quantitative and verbal scores, and scores from a personality profile quiz (http://www.truecolorspersonalitytest.com). Students were also oriented to the TBL process by reading ‘getting started with TBL’ handout (from the TBL collaborative website, http://www.teambasedlearning.org) and participating in a mock TBL class session.

### Statistical analysis

Each patient/client management course had two individual written exams (mid-term and final) and one individual practical exam at the end of the semester. A mean of these three exam scores, weighted equally, was used as the outcome measure to compare the effectiveness of the two instructional methods (traditional vs. TBL), presented as mean ±standard deviation. These mean exam scores were also converted into course letter grades (A: 90-100, B: 80-89.99, C: 70-9.99, and F<70) (Suppl. 1). We did not incorporate grades from RATs or peer evaluation, since these scores can inflate students’ final course grade.

The Shapiro-Wilk test of normality was performed on the exam scores for each cohort. The distributional shape of these scores was normal, justifying the use of parametric tests for further analysis. A one-way analysis of variance (ANOVA) was conducted to test the hypotheses of no significant difference in exam scores across years of student cohorts within the same instructional method for each course. Percentages of students receiving different letter grades for the two instructional methods were calculated. Independent sample t-tests were performed to compare the two instructional methods using the mean exam scores in both courses. Alternatively, an analysis of covariance would have been performed if significant differences in the mean UGPA between students of the two instructional methods were observed. Effect size was also computed. In addition, trend analyses were conducted to test for various trends of the mean exam scores across years of student cohorts. All hypothesis tests were conducted using two-sided tests with alpha set at 0.05. The IBM SPSS for Windows ver. 23.0 (IBM Corp., Armonk, NY, USA) was used to conduct all data analyses.

## Results

Baseline demographic and academic characteristics were similar for the two groups (traditional and TBL) in each course (Table 1) and there was no significant difference in UGPA beteween groups.

**Fig. 1.**
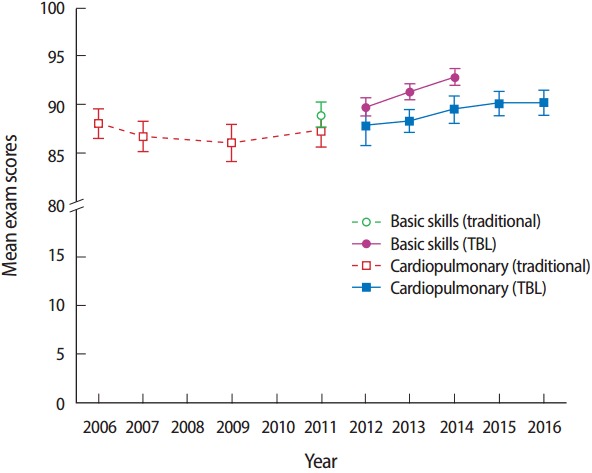
Mean of individual exam scores (with 95% confidential intervals) in two entry-level doctor of physical therapy patient/client management courses before and after implementation of TBL. TBL, team-based learning.

In the basic skills course, results of the one-way ANOVA indicated that there was a significant difference in the mean exam scores among the three years of student cohorts (2012-14) taught with the TBL approach (F(2, 147)=11.147, P<0.001). However, post hoc comparisons using the Scheffe test indicated that only the mean exam score of the 2012 cohort was significantly different from that of the 2014 cohort (P<0.05). Because the maximum difference in the mean exam scores between any two cohorts was only 3 points (out of 100), we treated these three years of student cohorts taught with the TBL approach as one group. When comparing the TBL method with the single year (2011) of traditional instruction in the basic skills course, there was a significant effect of instructional methods on the mean exam score (t(182)=3.629, P<0.001; Cohen’s d=0.69; confidence interval [CI], 0.31-1.07)); the mean exam score for the TBL group (91.3±3.4) was significantly higher than that of the traditional instructional method group (88.9±3.7). In addition, the trend analysis revealed that a significant linear trend was observed (F(1, 180)= 33.204, P<0.001), which indicated that as the years progressed from 2011 to 2014, the mean exam scores increased proportionately in a linear fashion (Fig. 1). There was also a significant linear trend in exam scores observed across the three consecutive cohorts (from 2012 to 2014) when TBL was implemented (P<0.001).

For the cardiopulmonary course, results of the one-way ANOVA indicated that there was no significant difference in the mean exam scores among the four cohorts (2006, 2007, 2009, and 2011) taught with the traditional instruction, or the five cohorts (2012-2016) taught with the TBL approach (F(3, 139)=0.986, P=0.401, and F(4, 220)=2.093, P=0.083, respectively). Given that no significant differences in the mean exam scores among cohorts in each instructional method in the cardiopulmonary course were observed, all cohorts receiving the same instructional method were treated as one group. There was a significant effect of instructional method on the mean exam scores in the cardiopulmonary course (t(366)=4.255, P<0.001; Cohen’s d=0.46; 95% CI, 0.24-0.67); the mean exam scores for the TBL group (89.3±4.8) was significantly higher than that of the traditional instruction group (87.0±5.2). In addition, the trend analysis revealed that a significant linear trend in the mean exam scores was observed (F(1, 359)=19.099, P<0.001) and indicated that as the years progressed from 2006 to 2016, the mean exam scores increased proportionately in a linear fashion across years (Fig. 1); no significant trend in exam scores was observed across the four cohorts (2006, 2007, 2009, and 2011) receiving traditional instruction (P= 0.471), but a significant linear trend was observed across the five consecutive cohorts (2012-2016) receiving TBL (P=0.007).

Finally, as indicated in Table 2, there was a 25% increase in students earning an ‘A’ across cohorts after switching from the traditional instructional method to the TBL approach in the basic skills course. For the cardiopulmonary course, there was an overall 15% increase in students earning an ‘A’ across cohorts after switching to the TBL approach, and a concomitant decrease in 10% earning a ‘B’ and 5% earning a ‘C.’

## Discussion

Extending the previous findings on the effectiveness of implementing TBL approach in improving PT students’ academic outcomes in gross anatomy courses [4-6], results of this study provided evidence to support the use of the TBL approach to increase students’ exam scores when compared to the traditional instructional method in two PT patient/client management courses. The effect sizes of the TBL approach on both courses were comparable, which are considered a medium effect. Other investigators also reported higher student exam scores when using TBL approach in patient/client management courses in other health profession curricula, including nursing, medicine, and pharmacy [8-10].

The increase in exam scores in DPT students taught using the TBL approach may be due to the following reasons. First, DPT students’ perceptions and satisfaction toward TBL have been reported to be favorable by other investigators [4-6], perhaps increasing stu dents’ engagement and interest in course content and therefore enhancing their learning. Second, several investigators reported students’ improved problem solving and increased knowledge retention when TBL approach was used [11,12]. All of these outcomes would likely increase exam performance. Future studies should include these learning outcomes when evaluating the effectiveness of TBL approach in patient/client management courses.

In addition to the increased mean exam scores, we also observed less variance in individual exam scores among student cohorts that were taught using the TBL approach as compared to the traditional instructional method (see error bars, Fig. 1). This decrease in exam score variance may be due to the fact that students showing lower academic performance benefit more from the TBL approach, especially since previous investigators found that the educational impact of TBL was greater for students in the lower academic quartile in a professional program, where ‘stronger’ students on a team are able to teach/coach ‘weaker’ students [13]. This benefit is a strength of TBL. Based on our experience, the tRAT and peer evaluation scores can inflate final letter grades. However, to eliminate this potential bias, we only included the scores from individual written and practical exams in our data analyses.

This study has several limitations. First, the study design lacked a control group and randomization. However, generalizability of the findings of this study was evidenced as the same results were observed in two different courses with two different instructors. A second limitation is that the basic skills course had only one year of traditional instruction with which to compare the TBL approach; however, the cardiopulmonary course had multiple years of traditional instruction to compare the TBL approach with, providing stronger evidence that TBL outperformed traditional instructional methods. In addition, GRE scores may have been an important covariate, but for various reasons they were not included in our analysis. These reasons included unavailability of percentile scores for all cohorts, inability to accurately impute percentile scores from raw scores, and changes in test format and score reporting over the 11-year period, all of which may invalidate any comparisons. Lastly, our learning outcomes were only evaluated in a single semester and did not assess long-term comprehension.

In conclusion, our findings suggest that using TBL in entry-level DPT patient/client management courses helped increase knowledge retention and problem solving (assessed via written exams) when compared to traditional instructional methods. Prior studies on TBL have shown that students perceived they had enhanced their teamwork and communication skills [14], skills deemed as ‘core competencies for interprofessional collaborative practice’ [15]. Thus, TBL has the potential to enhance patient outcomes through both improved knowledge retention and interprofessional collaborative skills. Future studies need to compare long-term learning outcomes, especially as related to patient-oriented outcomes, as well as satisfaction and perceptions of this instructional approach when implementing it patient/client management courses in a DPT curriculum.

## Figures and Tables

**Table 1.** Baseline demographic and academic characteristics of entry-level DPT students in two patient/client management courses before and after implementation of TBL

	Basic skills		Cardiopulmonary	
	Traditional instruction (N=34)	TBL (N = 150)	Traditional instruction (N = 143)	TBL (N = 225)
Gender (female)	23 (67.6)	105 (70.0)	115 (80.4)	160 (71.1)
Race/ethnicity (White)	33 (97.1)	138 (92.0)	128 (89.5)	209 (92.9)
Age (yr)	24 ± 2	23 ± 3	23 士 3	24 士 3
Overall UGPA)^a)^	3.59 士 0.27	3.64 士 0.23	3.60 士 0.27	3.65 士 0.23

Values are presented as number (%) or mean±standard deviation. Basic skills course: n = 34 in 2011, n = 50 in 2012-2014; cardiopulmonary course: n = 36 in 2006, n = 31 in 2007, n = 38 in 2009, n = 38 in 2011, n = 32 in 2012, n = 48 in 2013, n = 49 in 2014 and 2015, and n = 47 in 2016. DPT, doctor of physical therapy; TBL, team-based learning; UGPA, grade point average (undergraduate).

a) No significance difference (P > 0.05) on the mean UGPA was observed between traditional instruction and TBL in both courses.

**Table 2.** Course letter grade distributions^a)^ in two entry-level doctor of physical therapy patient/client management courses before and after implementation of TBL

Grade	Basic skills		Cardiopulmonary	
	Traditional instruction (N = 34)	TBL (N = 150)	Traditional instruction (N = 143)	TBL (N = 225)
A	44.1	69.3	31.5	46.7
B	55.9	30.7	59.4	49.3
C	-	-	9.1	4.0
				

Values are presented as %. TBL, team-based learning.

a) For the purposes of this analysis, results of the individual and team readiness assurance tests were not included in the course letter grade determination, only individual exam scores.

## Supplementary material

Supplement 1. Data file of the exam scores to DPT students at the University of Alabama.

Supplement 2. Audio recording of the abstract.
